# A Systematic Review and Meta-Analysis of Decision-Making in Offender Populations with Mental Disorder

**DOI:** 10.1007/s11065-018-09397-x

**Published:** 2019-02-23

**Authors:** Katy A. Jones, Thomas Hewson, Christian P. Sales, Najat Khalifa

**Affiliations:** 1 Division of Psychiatry and Applied Psychology, Room C24, Institute of Mental Health School of MedicineUniversity of Nottingham, Nottingham, UK; 2 School of Medicine, University of Nottingham, Nottingham, UK; 3 Nottinghamshire Healthcare NHS Foundation Trust, Nottingham, UK; 4 Department of Psychiatry, Correctional Service Canada, c/o Providence Care Hospital, Queen’s University, Kingston, ON Canada

**Keywords:** Decision-making, Offenders, Neuropsychological tasks, Iowa Gambling Task (IGT), Mental disorders, Personality disorder (PD), Meta-analysis

## Abstract

Decision-making has many different definitions and is measured in varied ways using neuropsychological tasks. Offenders with mental disorder habitually make disadvantageous decisions, but no study has systematically appraised the literature. This review aimed to clarify the field by bringing together different neuropsychological measures of decision-making, and using meta-analysis and systematic review to explore the performance of offenders with mental disorders on neuropsychological tasks of decision-making. A structured search of PubMed, Embase, PsycINFO, Medline, Cinahl was conducted with additional hand searching and grey literature consulted. Controlled studies of decision-making in offenders with evidence of any mental disorder, including a validated measure of decision-making were included. Total score on each relevant decision-making task was collated. Twenty-three studies met inclusion criteria (*n* = 1820), and 10 studies (with 15 experiments) were entered into the meta-analysis (*n* = 841). All studies included in the meta-analysis used the Iowa Gambling Task (IGT) to measure decision-making. Systematic review findings from individual studies showed violent offenders made poorer decisions than matched offender groups or controls. An omnibus meta-analysis was computed to examine performance on IGT in offenders with mental disorder compared with controls. Additionally, two sub-group meta-analyses were computed for studies involving offenders with personality disorder and psychopathy, and recidivists who were convicted of Driving While Intoxicated (DWI). Individual studies not included in the meta-analysis partially supported the view that offenders make poorer decisions. However, the meta-analyses showed no significant differences in performance on IGT between the offender groups and controls. Further research is required to ascertain whether offenders with mental disorder have difficulty in making advantageous decisions. An analysis of cause and effect and various directions for future work are recommended to help understand the underpinning of these findings. Trial Registration: CRD42018088402.

**Electronic supplementary material:**

The online version of this article (10.1007/s11065-018-09397-x) contains supplementary material, which is available to authorized users.

## Introduction

Decision-making is an integral part of our daily life. Understanding decision-making in people who commit crimes (hereby known as “offenders”) is particularly important because the decision to kill, assault, break into a house, steal, use substances, or break a court order has significant consequences for the offender, the victim of the offence, and wider society. Worldwide, the economic burden of crime is immense (e.g., see Institute of Economics and Peace, 2013). In the United Kingdom (UK) alone, there were 1.9 million incidents of violence recorded between 2012 and 2013 in England and Wales alone (Office for National Statistics, 2014), and the total cost of violence containment was estimated to be around £100 ($137) billion. There is a real-world need to understand why offenders make disadvantageous decisions.

### Defining and Measuring Decision-Making

There is currently no consensus definition for decision-making within the realm of neuropsychology, but attempts have been made to define it. For example, Gold and Shadlen (2007) define it as “a deliberative process that results in the commitment to a categorical proposition” (p.538). Ernst and Paulus (2005) posit decision-making reflects three distinct processes; namely the formation of preferences, selection and execution of actions, and evaluation of outcomes on choices. Others argue each of these processes is influenced by distinct, albeit overlapping, cognitive, affective and neural factors (Beszterczey, Nestor, Shirai, & Harding, 2013) as well as environmental or contextual factors (Ernst & Paulus, 2005). Indeed, from a neurobiological perspective, decision-making is thought to be influenced by the coordinated activity of a fronto-limbic network involving anterior prefrontal brain areas (particularly the lateral orbitofrontal cortex (lOFC) and the ventromedial frontal cortex (vmPFC)), the amygdala, the hippocampus, the pre-supplementary motor area, the striatum and the anterior cingulate cortex, as well as the dorsolateral prefrontal cortex (Bechara, Damasio, Damasio, & Anderson, 1994; Ernst & Paulus, 2005; Bechara, 2007; Beszterczey et al., 2013).

For the purposes of this review, decision-making was conceptualized in accordance with a framework proposed by Paret, Jennen-Steinmetz, and Schmahl (2017) whereby “individuals take into account the motivational value and probability of expected gains and losses in their decisions...in order to maximize outcomes, they weigh the options in hand according to the subjective value they attribute to each of these options” (page 302). Taking this approach ensures constructs such as decisions under conditions of uncertainty or risk, delay discounting, information sampling, and reinforcement reversal learning are included in the definition of decision-making. Table 1 summarises all neuropsychological tasks that could be considered to measure decision-making. Some tasks such as the Cambridge Gambling Task (CGT), Iowa Gambling Task (IGT), and Delay Discounting (DD) overlap with measurement of impulsivity, and thus have been used with those with SUD or gambling addiction (see Zois et al., 2014; Barry & Petry, 2008). Other measures of decision-making, such as in the Affective Decision-Making Task (ADMT, Ly, Huys, Stins, Roelofs, & Cools, 2014), include an affective component, where happy or angry faces are used as reinforcers or deterrents of behaviour. Other tasks such as The Ultimatum Game (UG) examine financial-based decision-making in a social context where players have to divide a sum of money.

**Table 1:** Summary of decision-making tasks

Task	Description of task
**Cambridge Gambling Task (CGT)** Rogers et al. (1999)	Individuals bet on the occurrence of either of two mutually exclusive events (whether a yellow token is hidden inside either a red or blue box) differing in their probability. Measures of performance include the quality of decision-making (i.e., the percentage of times subjects bet on the most likely outcome), the decision latency and the proportion of points bet.
**Iowa Gambling Task (IGT)** Bechara et al. (1994)	The IGT measures decision-making under conditions of uncertainty and risk. Individuals choose from four decks of cards, differing in terms of their reward-punishment profiles. Repeated selection from 2 of the decks (advantageous decks) results in overall net profit; whilst repeated selection from the 2 disadvantageous decks results in greater losses. The main measure of performance is the difference between the number of choices from the advantageous decks minus the number of choices from the disadvantageous decks, giving an overall ‘net’ score.
**Kirkpatrick et al. (** 2007 **) Borderline Personality Disorder Individual task** Rogers et al. (2003)	Participants choose between two simultaneous visually presented gambles – a control gamble and an experimental gamble. The control gamble always has an equal probability of winning and losing 10 points. Alternatively, the experimental gamble varies in its probability of winning and in the magnitude of its possible gains and losses. Outcomes are the proportion of choices of the experimental gamble over the control gamble as a function of its probability of winning, the sizes of possible gains and losses, and the mean deliberation times for the participants selections.
**Reasoning Ability Task (RAT)** Moritz et al. (2010)	Participants use probability estimates to decide whether they have sufficient information to warrant a decision. The main outcome measures for this task include the participants decision threshold (the lowest subjective probability at which a decision is made) and whether the participant demonstrates evidence of jumping to conclusions.
**Affective Decision-Making-Task** Ly et al. (2014)	Participants must learn through trial and error to approach or avoid different stimuli. Correct choices are reinforced probabilistically, being more likely to result in a monetary reward. At the start of each trial, the participant is presented with a task-irrelevant angry or happy face. This assesses for emotional biasing of instrumental action, whereby angry faces provoke instrumental avoidance.
**Behavioural Investment Allocation Strategy Task** Khunen and Knutson (2005)	In this task, participants must make a series of selections between 2 stocks and 1 bond. Bonds always return a small profit, whereas stocks win or lose larger sums based on pre-determined probabilities. This task has been used to analyse neural activity associated with uncertainty and risky decision-making.
**Ultimatum Game (UG)** Güth, Schmittberger, and Schwarze (1982)	The UG is a model of economic decision-making. It involves one participant dividing a sum of money between themselves and another player, who must then accept or reject the proposed offer. Accepted offers are enacted, whilst rejected offers result in both players receiving nothing. Rational responders should accept every positive offer, since there are no additional rounds with the same opponent to encourage behaviour change; however, actual responders often decline unfair offers, allowing their emotions to override economical rationality.
**Secret Agent (SA)** Young, Gudjonsson, Goodwin, Perkins, and Morris (2013)	In this task, the participant must move along a series of game-boards by choosing low, medium or high-risk pathways. They are also faced with a number of moral dillemas along their trajectory. Their choices are scored using risk-taking and moral decision-making subscales, allowing measurement of the extent of their risk-taking and empathy and prosocial behaviour.
**Individual computer-based delay discounting task** Jones et al. (2015)	Participants must make choices between paying/receiving a given sum of money immediately, or paying/receiving a different amount after a stated time delay. Most individuals show a preference for smaller-sooner rewards rather than waiting for a temporally delayed better outcome. The main outcome measure for this task is the indifference point, which is the point at which the subject finds it difficult to decide between the immediate and delayed rewards/losses.

### Decision-Making in Offenders

There has been much speculation about the decision-making ability of offenders (Yechiam et al. 2008). Drawing on criminological theory, both low levels of self-control and impairment in rational choice are popular explanations for why offenders make the decision to commit a crime (Piquero & Tibbetts, 1996). Rather than focusing on individual characteristics of offenders, theories of offending such as Routine Activity Theory (Cohen & Felson, 1979) placed importance on the situational context, such as the opportunity to commit a crime, and the role of economic deprivation in influencing criminal behaviour. Conversely to this, the Rational Choice Theory (RCT) of offending applied to criminology argued that a person who commits a crime is an active agent who makes a series of decisions which will include considering the (sometimes limited) information available to them (Cornish & Clarke, 1987; Cornish & Clarke, 2014). If agreeing with the RCT, developing an understanding of the decision-making ability of offenders, including the threshold for risk, availability and use of information, and ability to resist immediate reward, would be important factors to measure and understand. As such, there is a merit in understanding what may drive decision-making in an offender group using neuropsychological tasks, as they have been found to have some relation to real-life offending behaviour (Beszterczey et al., 2013).

Understanding decision-making specifically in offenders with mental disorder is important for several reasons. Firstly, from an epidemiological perspective this is a significant issue; the UK prison population alone exceeded 84,000 persons in December 2017 (UK Government Statistics, 2017) and evidence exists for higher rates of mental disorder among prisoners than in the general population (Singleton, Farrell, & Meltzer, 1999). The strongest and most consistent evidence is for the prevalence of psychosis, substance use disorders (SUDs) and depression in prison (Fazel, Hayes, Bartellas, Clerici, & Trestman, 2016). For example, a systematic review of severe mental disorder in prisoners worldwide showed that one in seven people in prison had a diagnosis of either major depression or psychosis (Fazel & Seewald, 2012). Personality disorders are also prevalent amongst offender groups such as prisoners (Singleton et al., 1999). Secondly, nearly 60% of people released from prison re-offend within three years of their release (Pager, 2003) and evidence indicates that mental disorders substantially increases a person’s risk of violent reoffending (Chang, Larsson, Lichtenstein, & Fazel, 2015). Consequently, the societal impacts of criminal decision-making are particularly large and recurrent in this offender subgroup. Thirdly, the majority of previous research has tended to focus on offenders and persons with mental disorder separately; this means that little is known about the combined effects that these two factors have upon decision-making. Finally, this topic has significant clinical, legal and societal implications. If we begin to identify specific deficits in decision-making that predict offending behaviour amongst individuals with mental disorder, such deficits could inform risk assessment and be targeted as part of treatment and rehabilitation. This would improve patient care and reduce rates of recidivism and harm to society, whilst also reducing the burden on our penal systems.

### The Present Review

Though studying the underlying decision-making processes in all offender groups is beyond the scope of this review, we aim to review decision-making in offenders with mental disorder. While work has been done to review decision-making separately for some different types of mental disorder (e.g., Hughes, Dolan, & Stout, 2016), results remain inconclusive. For example, when considering psychopathy alone, three studies (Blair, 2001; Boulanger, Habib, & Lancon, 2008; Mitchell, Colledge, Leonard, & Blair, 2002) concluded people with psychopathy made less advantageous choices than those without psychopathy, possibly due to interference in affective processing. However, an equal number of studies found no significant difference between psychopathic and non-psychopathic individuals (Blair & Cipolotti, 2000; Lösel & Schmucker, 2004; Schmitt, Brinkley, & Newman, 1999). Hughes et al. (2016) concluded the differences in sample and setting could account for this inconsistency. They concluded evidence needs to be pooled for IGT in psychopathy and the issue needs to be investigated further. This present review aims to fulfil these recommendations, whilst also examining the effects of other forms of mental disorder upon offender decision-making.

## Aims

We aimed to conduct a systematic review and meta-analysis of the literature on decision-making in offenders with mental disorder. A meta-analysis is important in order to pool results of studies to establish whether offenders with mental disorder overall make poorer decisions than controls. As these populations are often complex with multiple comorbidities, the systematic review adds context to individual studies. To our knowledge no meta-analysis or systematic review of this literature has been conducted to date, thus this is the first meta-analytic review bringing together the neuropsychological literature in this group.

## Method

### Protocol and Registration

The protocol for this review has been registered on Prospero (registration number: CRD42018088402).

### Inclusion Criteria

Eligibility criteria were determined using the PICO framework, and studies were included in the review if they met the following criteria: participants were offenders (defined by having committed any crime) with any diagnosis of mental disorder including SUDs who were assessed using any well-validated measure of decision-making (“Intervention”). The term “mental disorder” was chosen based on the World Health Organisation (WHO) definition as an umbrella term which describes a broad range of disorders. According to the WHO, “mental disorders” are characterized by “a combination of abnormal thoughts, emotions, behavior and relationships with others” (WHO, 2018, P.1). The extent to which the task was considered a measure of decision-making was based on the framework of decision-making outlined in the introduction, and in Table 1. When it was unclear whether the task was a measure of decision-making, the study was excluded. Samples were checked to ensure participants were not intoxicated during the time of the study. Papers that included participants with Traumatic Brain Injury (TBI) were excluded as reviewers felt this would present an additional challenge when interpreting the results. Consideration was given to inclusion of studies involving people with sub-threshold symptoms of mental disorder. This is relevant in the area of SUDs since many studies examine individuals who misuse substances at meaningful levels but may not meet clinical criteria for SUD (i.e., heavy drinkers, smokers, frequent gamblers). The control group could be either offenders without a diagnosis of mental disorder or non-offenders. Outcome measures were relevant scores on decision-making tasks. Expert opinion papers and editorials were excluded. Otherwise, there were no other exclusion criteria for study design. All studies had to be published in the English language.

### Search Strategy

An electronic database search was conducted on 24th July 2018 for the following databases: PubMed (searched to 2018), PsycINFO (1806 to 2018), Medline (1946 to 2018), Embase (1974 to 2018), and Cinahl (1981 to 2018). The following search terms were used: Decision-making (“decision-making” OR “gambling task” OR “temporal discounting” OR “delay discounting” OR “deferred gratification” OR “intertemporal preference” OR “model based”) AND mental disorders (“mental disorders” OR “schizophrenia” OR “personality disorder” AND “schizo” OR “mental” OR “psych” OR “personality disorder” OR “depression” OR “anxiety”) AND offenders (“criminals” OR “prisoners” OR “forensic psychiatry” OR “Offend” OR “Felon” OR “Convict” OR “Delinquent” OR “Prison” OR “Criminal” OR “Jail” OR “Remand” OR “Imprison” OR “Detention” OR “Correctional Facility” OR “Probat” OR “Inmate” OR “Juvenile Delinquent” OR “Forensic” OR “Sentenced” OR “Detainee”). Only studies published in English were included, therefore “English” was included in each search.

Additional hand searching was conducted by one review author (TH) by close reading included studies to identify any other eligible material. Grey literature was explored.

### Data Extraction and Statistical Analysis

Following the initial search, deletion of duplicates, article title, and abstract screening was conducted by one review author (TH), and full text of remaining studies was reviewed by the same author. The whole review team (KJ, TH, CS, and NK) reviewed all identified eligible studies and made decisions about exclusion based on criteria described in the previous section.

A standardized template was used to collect study data for all eligible articles. The following information was extracted: author (date), title, country, methods (design), participants (sample size, gender, age, ethnicity), inclusion and exclusion criteria, baseline characteristics (including psychotropic medication), setting, decision-making task, primary outcome measure (total score on decision-making task), and secondary outcomes (scores on other relevant tasks, IQ, recidivism, and substance use) and additional notes for each study.

### Summary Measures

In the original planned analysis, we intended to examine each decision-making task separately. However, after examination of the literature, only studies involving the IGT provided sufficient data for meta-analysis. Therefore, summary statistics were expressed as the difference in means between the groups with 95% confidence intervals (CIs). The main effect was recorded as a negative value if the effect was in the predicted direction (e.g., favouring offenders with mental disorder groups) and as a positive one if it was in the opposite direction. Each experiment was used as the unit of analysis to obtain differences in means in the meta-analysis. For studies involving more than one control group or condition, only the comparison between experimental and control group (condition) was selected. The meta-analyses were conducted using Review Manager Version 5.3 (2014).

### Synthesis of Results & Measures of Inconsistency

Meta-analyses with the random-effects model were performed to assess decision-making abilities overall and by diagnosis. Heterogeneity between the studies was assessed using I^2^ and T^2^ statistics (Higgins & Thompson, 2002; Higgins, Thompson, Deeks, & Altman, 2003). I^2^ is a measure of relative heterogeneity. I^2^ estimates the percentage of the variability in effect estimates that is attributable to heterogeneity rather than chance. An I^2^ value of greater than 40% indicated moderate heterogeneity, and a value greater than 60% indicated high heterogeneity (Deeks, Higgins, & Altman, 2008). T^2^ provides an estimate of absolute heterogeneity. When T^2^ increases, the observed variance increases or the variance within-studies decreases (Borenstein, Hedges, Higgins, & Rothstein, 2017)***.***

### Risk of Bias - Publication Bias

A funnel plot was produced for the analysis of all included trials (See Online Figure 1). Among studies reporting decision-making in offender populations with mental disorder, no evidence of publication bias was present based on Begg and Mazumdar (1994)’s (*p* = .22) and Egger, Smith, Schneider and Minder (1997)’s (*p* = .09) tests. Funnel plots are not recommended if fewer than 10 studies are inputted for analysis (Sterne et al., 2011), thus funnel plots were not produced for sub-group analysis. As the tasks were broadly consistent, it may be the variable clinical population of the participants used that is accounting for the differences observed across studies.

### Quality Assessment of Included Studies

All studies were assessed using the NIH Quality Assessment Tool for Observational, Cohort and Cross-sectional Studies: https://www.nhlbi.nih.gov/health-pro/guidelines/in-develop/cardiovascular-risk-reduction/tools/cohort. Each study was assessed against several criteria. The total number of criteria that each study achieved, and the potential impacts that not achieving particular criteria would have had upon each study’s results, were used to form an overall quality rating for each study (good, moderate or poor).

## Results

**Fig. 1:**
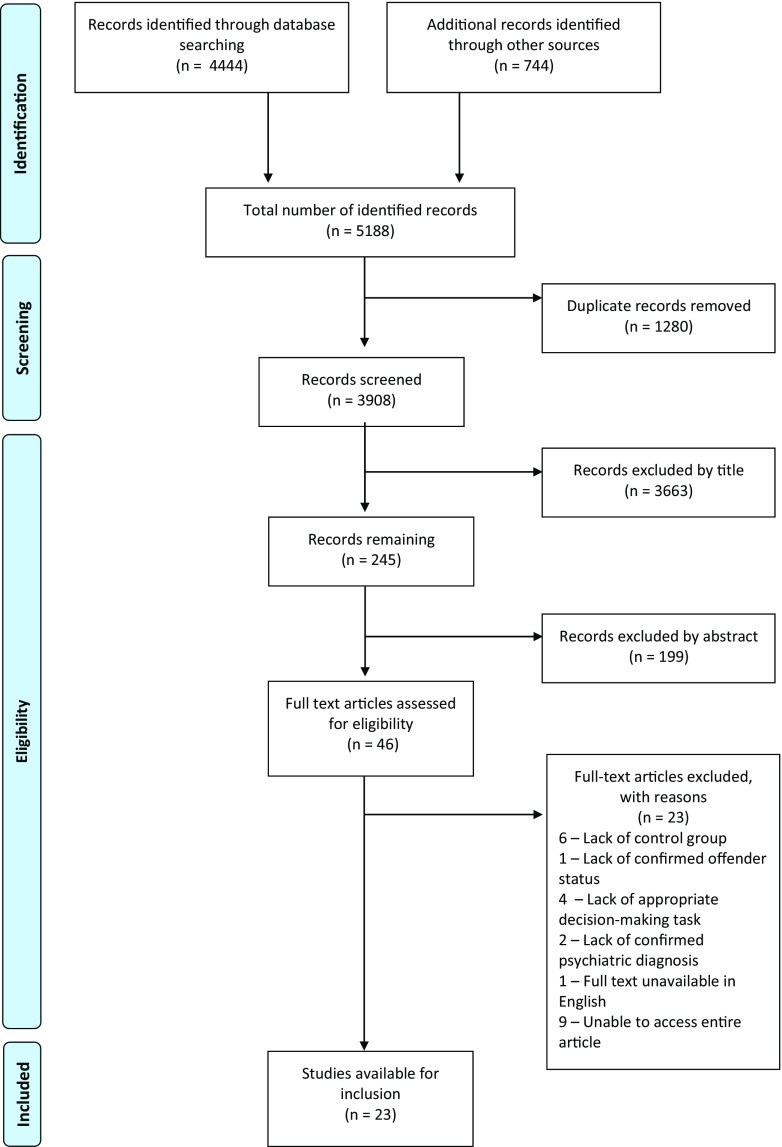
PRISMA Flow Diagram

A total of 4444 records were identified through electronic database searching, with 744 identified through other sources (see Fig. 1). After removal of duplicates, 3908 articles were screened. 46 remained following exclusion of articles by title and abstract; the full English language text was not available for 10 of these, therefore 36 full text articles were assessed for eligibility. 13 articles were excluded (see Online Table 1 for a brief description of each and reasons for exclusion), leaving 23 articles for inclusion. Table 2 presents a summary of all 23 included studies (Total *n* = 1820 including 1039 patients and 781 controls) organized by task type. We were able to extract data from 10 studies (with 15 experiments) to enter into the meta-analysis (Total *n* = 841, including 426 patients and 415 controls). Although eligible for inclusion, three studies using the IGT (Beszterczey et al., 2013; Kolla et al., 2015; Wells & Brown, 2014) were not entered into the analysis available despite multiple attempts to contact the authors. Studies entered into the meta-analysis are indicated with an asterisk (Table 2). Main results of 13 other eligible studies were qualitatively summarized.

**Table 2:** Characteristics of all included studies that assessed decision-making in an offending population

Study (Year) Country	Design	Setting	Offenders n (% male, mean age in years + SD)	Disorder type	Controls n (% male, mean age in years + SD)	Control type	Task
Beszterczey et al. (2013) USA	Case-control	Offender re-entry programme	26 (100%, 36.52 + 7.57)	PD - Psychopathy	30 (100%, 27.40 + 7.66)	University students	IGT- 4 deck
Bouchard et al. (2012)* Canada	Case-control	Douglas Mental Health University Institute (Psychiatric hospital)	23 (87%) IGT-R Hi (44.2 + 10.59) IGT-R Lo (44.15 + 10.02)	DWI	19 (89.5%, 40.79 + 9.00)	Non-offenders	IGT- 4 deck
Broom (2011)* Canada	Case-control	3 Correctional Facilities serving offenders on remand or with sentences <2 years	67 (100%, 33.22 + 10.17)	PD- Psychopathy (N = 18/67)	65 (29%, 18.87 + 1.78)	University students	IGT- 4 deck
Brown et al. (2016)* Canada	Between-subjects	Douglas Mental Health University Institute (Psychiatric Hospital)	36 DWI (100%, 30 + 5.7) 28 SPEED (100, 28.7 + 5.0) 27 MIXED (100%, 27.8 + 6.1)	DWI	47 (100%, 30.1 + 6.2)	Low risk drivers	IGT- 4 deck
Gulec (2007)* Turkey	Case-control	Prison	47 (100%, 25.21 + 8.67)	ADHD	69 (100%, 37.06 + 13.80)	Prisoners no ADHD	IGT- 4 deck
Hughes, Dolan, Trueblood, & Stout (2015)* Australia	Case-control	Prison (maximum and low to moderate secure)	60 (100%, 36 + 11.81)	PD- Psychopathy	20 (100%, 30 + 11.09)	Non-offenders	IGT- 4 deck
Kasar, Gleichgerrcht, Keskinkilic, Tabo, & Manes (2010)* Turkey	Case-control	Specialist treatment programme (Traffic education)	34 (100%, 35.4 + 8.4)	DWI	31 (100%, 35.1 + 9.5)	Healthy controls	IGT- 4 deck
Kolla et al. (2015) Canada	Case-control	Community and probation services	18 (100%, 36.2 + 9.4)	PD - ASPD	18 (100%, 36.4 + 8.9)	People without ASPD	IGT- 4 deck
Nishinaka et al. (2016)* Japan	Case-control	3 Forensic hospitals	71 (85%, 42.79 + 11.92)	Psychosis (1 with mood disorder)	54 (89%, 42.06 + 11.43)	Healthy community volunteers	IGT- 4 deck
Rodriguez & Ellis (2018)* Australia	Case-control	Specialist treatment programme (Sex offender)	11 FTCEMO (100%, 61.0 + 7.8) 34 HSO (100%, 62.3 + 6.6)	Paedophilia	32 (100%, 57.3 + 6.5)	Offenders with no sex-offender history	IGT 4-deck
Sedgwick (2017)* UK	Between-subjects	Forensic hospital (high-secure)	15 Psychotic disorder no DPD (100%, 35.8 + 7.99) 17 DPD only (100%, 37.4 + 10.9) 26 DPD and psychosis (100%, 36.7 + 9.39)	PD- DPD and Psychosis	30 (100%, 39.3 + 10.4)	Healthy controls	IGT- 4 deck
Wells & Brown (2014) Canada	Case-control	Douglas Mental Health University Institute (Psychiatric Hospital)	27 High risk drivers (100%, 29.04 + 5.02)	DWI	15 (100%, 24.80 + 4.71)	No moving violations or DWI convictions	IGT- 4 deck GDT
Young et al. (2013)* UK	Case-control	Forensic hospital (high secure)	50 severe mental illness (100%, 35.40 + 9.49) 50 PD (100%, 39.24 + 9.96)	PD and Psychosis	50 (100%, 38.06 + 12.74)	Healthy volunteers	IGT- 4 deck SA
Baliousis (2014) UK	Between-subjects	Forensic hospital (medium secure)	52 ASPD (100%, 30.3 + 8.9) 33 no ASPD (100%, 37.8 + 9.2)	PD- ASPD	20 (100%, 33.9 + 10.7)	Healthy volunteers	CGT
DeBrito, Viding, Kumari, Blackwood, & Hodgins (2013) UK	Between-subjects	Probation Services	17 ASPD+P (100%, 40.0 + 9.0) 28 ASPD-P (100%, 35.8 + 8.4)	PD-ASPD ± Psychopathy	21 (100%, 35.0 + 8.2)	Healthy non-offenders	CGT
Jones, Fearnley, Panagiotopoulos, & Kemp (2015) Australia	Case-control	New South Wales Drug Court	80 (85%, 32.5)	Substance abuse	101 (31%, 19.1)	University students	DD (computer and paper)
Kirkpatrick et al. (2007) UK	Case-control	Prison (high and medium secure)	17 (100%, 34.65 + 7.27)	PD - BPD	17 (100%, 38.35 + 9.29)	Offenders with PD but not BPD	BPD Individual Task
Kuokkanen, Lappalainen, Repo-Tiihonen, Tiihonen, & Aho-Mustonen (2016) Finland	Cross-sectional, pilot	Forensic hospital (high secure)	10 (100%, 43.55 + 12.24)**	Schiz	10 (100%, 43.55 + 12.24)**	Non-offenders with schizophrenia	RAT
Ly et al. (2016) Netherlands	Case-control	Forensic hospital (high-secure)	38 (100%, 37.7 + 9.6)	PD	19 (100%, 44.7 + 10.7)	Staff at high secure hospital	ADMT
Prehn et al. (2013) Germany	Case-control	Forensic hospital (high secure)	11 ASPD with EHO (100%, 27.55 + 5.57) 12 ASPD with EHE (BPD) (100%, 27.75 + 10.24)	PD – ASPD ± BPD	13 (100%, 26.62 + 8.40)	Healthy controls	BIAS task
Koenigs, Kruepke, & Newman (2010) USA	Between-subjects	Prison (medium secure)	6 primary psychopaths (100%, 30.3 + 9.4), 6 secondary psychopaths (100%, 30 + 4.9)	PD- Psychopathy	22 (100%, 35.1 + 6.9)	Offenders who without psychopathy	Ultimatum Game (paper-based)
Radke, Brazil, Scheper, Bulten, & De Bruijn (2013) Netherlands	Between-subjects	Forensic hospital (high secure)	18 with psychopathy (100%, 42.5 + 6.7) 14 no psychopathy (100%, 39.7 + 7.7)	PD- Psychopathy	18 (100%, 37.4 + 8.8)	Healthy controls	Ultimatum Game (computerised)
Young, Gudjonsson, Carter, Terry, & Morris (2012)* UK	Case-control	Medium secure and closed ward	30 (26 psychosis, 4 PD) (100%, 38.7 + 12.9)	PD and Psychosis	60 (100%, 37.9 + 13.4)	Healthy community volunteers	SA

*IGT* Iowa Gambling Task, *IGT-4* 4 deck, *IGT-5* 5 deck, *SA* Secret Agent Task, *DD* Delay Discounting, *RAT* Reasoning Ability Task, *CGT* Cambridge Gambling Task, *ADMT* Affective Decision-Making Task, *GDT* Game of Dice Task, *PD* Personality Disorder, *BPD* Borderline Personality Disorder, *DPD* Dependent Personality Disoder, *DWI* Driving whilst influenced, *Schiz* Schizophrenia, *ASPD* Anti-Social Personality Disorder, *ADHD* Attention Deficit Hyperactivity Disorder, *SPEED* Non-alcohol reckless drivers, *MIXED* Drivers with mixed risk-driving profile, *IGT-R Hi* Subgroup of offenders with IGT scores above the median value for all offenders, *IGT-R Lo* Subgroup of offenders with IGT scores below the median for all offenders, *FTCEMO* First Time Child Exploitation Material Offenders, *HSO* Historical Sex Offenders, *EHO* emotional hypo-reactivity, *EHE* emotional hyper-reactivity

*Studies were entered into meta-analysis

**Demographics for forensic and non-forensic patients are not given separately. The numbers shown are representative of the entire sample (cases and controls)

Table 2 shows eighteen studies recruited males only in both their offender and control groups and the remaining four showed some diversity of gender within the sample. Studies included represented a number of different countries including six from the UK, three from Australia, two from the Netherlands, one from Germany, two from the USA, one from Finland, and one from Japan. Study setting varied with twelve in low, medium or maximum secure hospitals, four in prison, and six in community or probation services. Some community programmes included specialist education such as sex offender or traffic education. One study included offenders attending a drug court (Jones et al., 2015). Disorder type varied and included personality disorder (psychopathy, borderline personality disorder [BPD], and dissocial personality disorder [DPD]), psychosis, and attention deficit hyperactivity disorder (ADHD), and Paedophilia. Three of the included studies investigated drinking whilst under the influence of alcohol (DWI) behaviour in recidivists which as well as being an offence, we also considered to be a proxy of sub-threshold SUD.

The majority of control samples included healthy controls with no offending history recruited through the community or university. Other examples of control groups were low-risk drivers, or those without any DWI convictions, non-offenders with a mental disorder, offenders without a mental disorder and staff working at a secure hospital. One study used a three-group comparison design with gradients of psychopathy (Koenigs et al., 2010). The majority of included studies used the IGT to measure decision-making. All these studies used the 4-deck variant of the IGT. Two studies used the CGT, one computer and paper-based DD, one used the BPD individual task, one used the RAT, one used the ADMT, one the BIAS task, two the UG, and two the SAT.

### Quality of Studies

Table illustrating study quality is available online (Online Table 2) and shows the majority of the studies were of moderate quality, with two being rated as good (Baliousis, 2014; Brown et al., 2016) and a further two being rated as poor (Gulec, 2007; Wells & Brown, 2014). Controls were frequently well matched to offenders with mental disorder on a wide range of sociodemographic variables. Whilst some studies excluded participants who were being prescribed psychotropic medications, the impact of medications and comorbid diagnoses on decision-making capabilities was generally poorly assessed. All but one of the studies (Jones et al., 2015) were cross-sectional in nature. This made it difficult to establish causal relationships and to examine how decision-making capabilities may have changed over time and during a patient’s treatment and/or time in custody.

### IGT Performance in Offenders with all Types of Mental-Disorder

**Fig. 2:**
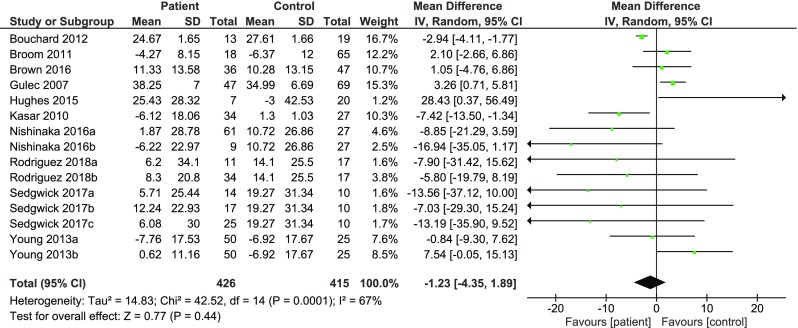
Overall meta-analysis decision-making in offenders using IGT

Fifteen experiments were entered into the overall meta-analysis to examine decision-making on the IGT (4-deck) in offenders with different types of mental disorder. Figure 2 shows the results of the meta-analysis (See Online Figure 1 for funnel plot). The overall effect was not statistically significant (MD = −1.23, 95% CI, −4.35 to 1.89). High heterogeneity was observed across all the studies (T^2=^ 14.83 *X*^2^ = 42.52, df = 14, *p* = 0.001; I^2^ = 67%). Two separate meta-analyses were conducted for on Psychopathic disorders and PD as well as DWI which was used as a proxy for SUD.

### Decision-Making in Offenders with Psychopathic Disorder and PD

Four studies were entered into the meta-analysis (See Online Figure 2). The overall effect was not statistically significant (MD = 1.74, 95% CI, −4.74 to 8.22). Low heterogeneity was observed across all the studies (T^2^ = 14.29 *X*^2^ = 4.45, df = 3, *p* = 0.22, I^2^ = 33%).

### Decision-Making in Offenders Who Were DWI

Three studies were entered into the meta-analysis (See Online Figure 3). Two studies favored offender group, offenders made poorer decisions than controls (Bouchard, Brown, & Nadeau, 2012; Kasar et al., 2010) with one study favoring control (Brown et al., 2016). The overall effect was not significant (MD = −2.99, 95% CI, −6.32, 0.34). Moderate heterogeneity was observed across the studies (Tau^2^ = 4.60, Chi^2^ = 3.89, df = 2, *p* = 0.14, I^2^ = 49%).

### Summary of Studies Not Entered into Meta-Analysis

All studies not entered into the meta-analysis will be reviewed here. Studies are reviewed by task type.

### IGT

Beszterczey et al. (2013) found that compared to controls, recidivists with psychopathy were unable to learn from feedback on the IGT, characterized by their inability to modify their preferences to more advantageous decks of cards. Higher levels of psychopathy, as characterized by high scores on the Hare Psychopathy Checklist-Revised HCL-R score (Hare, 2003), were related to poorer decisions. In a small sample of male offenders with ASPD, Kolla et al. (2015) found that orbitofrontal cortex (OFC) and low brain monoamine oxidase-A total distribution volume (MAO-A VT) were lower in ASPD and that IGT performance was negatively correlated with VS MAO-A VT, meaning the lower the VS MAO- A VT, the more risky decision-making on the IGT.

Wells and Brown (2014) explored IGT and GDT performance in 27 high-risk drivers (HRDs) who had had at least three moving violations e.g. speeding in the last two years or had 2 or more DWI convictions. Control group (*n* = 25) included people with no moving violations or DWI history. HRDs did not differ from control participants in decision-making on the IGT or the GDT. Sub-group analysis was conducted by dividing HRDs into those who had DWI involvement or not. Those who had not been convicted of DWI performed better on the IGT and GDT compared to CTLs and HRDs who have been convicted of DWI. However, due to small sample size, these results should be treated with caution.

### CGT

DeBrito et al. (2013) examined executive functioning in violent offenders with ASPD and psychopathy (*n* = 17), violent offenders with ASPD without psychopathy (*n* = 28), and non-offenders (*n* = 21). They used the CGT to assess decision-making. Results showed that both groups of offenders, compared to non-offenders, made poorer quality decisions. All groups increased deliberation times as the box ratio became less favorable, demonstrating the offender groups were aware of the changing probabilities and increased risk of losing points but did not adjust their behaviour. They related this to real life decision-making, whereby antisocial behaviour is continued, despite an awareness of the negative consequences. Lack of between-group differences for offenders in this study suggests those with ASPD alone, and those with ASPD and psychopathy show similar patterns of decision-making.

Also exploring CGT performance, Baliousis (2014) found both patients with ASPD, and with personality disorders other than ASPD, performed significantly worse than controls on the CGT overall; however, the deficits in decision-making emerged in easier conditions for the ASPD group. The offenders with ASPD additionally demonstrated impairments in tasks of motor regulation, response reversal, risk-taking, working memory and visual perception, but such deficits weren’t found to exist in the group of offenders with other PDs. Patients with psychopathy demonstrated significantly poorer decision-making than controls in only the ascending and 7:3 odds conditions of the CGT. In contrast, those with PDs other than psychopathy performed significantly worse across almost all conditions of the CGT. Specific deficits in the psychopathy group were elicited in tasks of response reversal and visual perception, whilst the offenders with other PDs experienced greater difficulties in tasks of planning, attentional set-shifting, and visual short-term memory.

### DD

Jones et al. (2015) used the DD task to measure decision-making, but they referred to this as impulsivity in the manuscript. They compared 80 participants attending an Australian drug court with 101 university students (non-offenders). The majority of offenders (78.8%) had a history of burglary or theft. Recent drug use histories (last 3 months) included opioids (73.8%) and amphetamine (70%). Results showed offenders had higher discount rates (were more impulsive) than controls. Drug court participants discounted delayed gains more than delayed losses.

### BPD Task

Kirkpatrick, Joyce, Milton, and Duggan (2007) used a decision-making task originally developed by Rogers et al. (2003) to measure emotional decision-making. They compared offenders (*n* = 17) with histories of serious violent or sexual offences and a diagnosis of DSM-IV (American Psychiatric Association, 1994) BPD, with controls (17 participants with similar offending histories, and personality disorder diagnoses, but no diagnosis of BPD). This simple gambling task (described in Table 1) included the following dependent measures: proportion of choices of the experimental gamble over the control gamble (proportional choice) as a function of probability of winning, sizes of gains and sizes of losses and mean deliberation time (in milliseconds) for these choices. Offenders with BPD engaged in more risky options than controls, exhibiting deficits in processing of information about potential loss (punishment) when the probability of gains (reward) was high. Authors argued that a diagnosis of BPD and a history of serious offences result in problems integrating different reinforcement signals when choosing between risky actions.

### RAT

Kuokkanen et al. (2016) conducted a pilot study using the RAT task with 20 male inpatients with schizophrenia, 10 of which had a history of violence. Using the draws-to-decision (DTD) variant of the Jumping to Conclusions (JTC) paradigm as the outcome variable, they found 75% of patients with schizophrenia made hasty decisions based on a limited information. Information gathering in the task was related to clinical insight and distress, with more information gathered related to more insight, and less distress, although authors acknowledged that the effect could be the other way around. Authors related poor decision-making to schizophrenia symptoms and lack of insight. Although half the sample had violence in their history, they did specifically explore the role of this in decision-making.

### ADMT

Using the ADMT, Ly et al. (2016) examined affective decision-making in 37 violent offenders with a history of psychiatric disorders and 19 controls with no criminal record or history of mental disorder. Results showed control participants avoided angry faces, but violent offenders did not. Authors argued this result shows the consequences of disordered affective processing for instrumental action, particularly in those who commit violent offences and score highly on psychopathic traits. In clinical terms, this may explain why people with a history of violent offending may not be affected by typical social-emotional cues (e.g., screaming or crying) that would or interfere with their decision to commit the act.

### BIAS

Using the BIAS task Prehn et al. (2013) asked two groups of violent offenders, and one group of controls to choose between low and high-risk financial options (bonds vs. stocks) whilst undergoing functional magnetic resonance imaging (fMRI). The emotional hypo-reactive offender group (EHO, *n* = 11) were selected by using cut off scores for psychopathy, and no presence of BPD, while the emotional hyper-reactivity offender group (EHE, *n* = 12) fulfilled the criteria for BPD, but not for psychopathy. Healthy controls (*n* = 13) had no history of offending, nor of psychopathy or BPD. Findings showed that the EHO offenders performed significantly worse on the task in comparison to controls, and they also showed diminished neural activation in the rostral anterior cingulate cortex (rACC) during times of uncertainty in the task. Prefrontal cortex (PFC) changes in the EHO group related to decreased activity in this area when attempting to regulate their behaviour. Authors concluded that those with psychopathic traits are less able to emotionally represent uncertainty and to anticipate punishment when making decisions.

### UG

Radke et al. (2013) examined decision-making in offenders (unclear as to offender “type”) with psychopathy using the UG. Male offenders with (*n* = 18) and without psychopathy (*n* = 14) were compared to healthy controls (*n* = 18). Results showed that offenders without psychopathic traits showed deficits in social decision-making, while offenders with psychopathy did not differ from controls. Koenigs et al. (2010) explored performance on the paper-based UG with 47 male prisoners in a medium secure correctional institution. They used the Psychopathy Checklist Revised (PCL-R) and Welsh Anxiety Scales (WAS) to screen participants for the presence of psychopathy. Unlike the study above, a three-group comparison design was used with participants separated into “primary psychopaths” (scoring 30 or greater on PCL-R and 13 or less on WAS), “secondary psychopaths” (scoring 30 or greater on PCL-R and 14 or greater on WAS), and “non-psychopaths” (scoring 20 or less on PCL-R). Results showed deficits in decision-making for primary psychopaths as indicated by lower acceptance of unfair offers compared to secondary and non-psychopaths. There were no significant differences between secondary psychopaths and non-psychopaths on UG performance. Authors suggests such a pattern of responding for primary psychopaths mirrors those with vmPFC lesions.

## Discussion

This meta-analytic review aimed to collate all studies of decision-making in offenders with mental disorder, exploring type of task typically used to measure decision-making and type of disorder represented. Findings did not support the suggestion that offenders (with different types of offence history) who have mental disorder make poorer decisions than controls. The most common decision-making task used with this population was IGT (4-deck variant only), and the meta-analysis of these studies showed non-significant results. Additionally, results from an overall meta-analysis of decision-making including studies using the SAT were also not-significant. Due to limited studies, it was not possible to enter outcome measures from other popular decision-making tasks such as the DD or CGT into the meta-analysis, nor was it possible to include tasks used less commonly in a forensic population (e.g., the UG). Findings from individual studies in the systematic review suggest that decision-making (as conceptualized by that individual task), was poorer in offenders with different mental disorders, furthermore, individual studies pointed to specific deficits in decision-making for each specific mental disorder. Overall, the decision-making functions of offenders with mental disorder remains broadly unclear, with abnormalities in decision-making processing being identified but no statistically significant effect on overall decision-making function being elicited. This review has highlighted a number of methodological considerations and directions for future work to help us better understand this important topic.

With the exception of Wells and Brown (2014) who tested decision-making in those who were high risk drivers, and Jones et al. (2015) who used a sample of those with drug use offences, the majority of reviewed studies used samples of violent offenders, and in most cases, who show psychopathic traits. Such studies of violent offenders with mental disorder tended to consistently find impairments in decision-making, although this does not support the overall meta-analysis of the larger offender cohort. Individual studies that did find an effect showed that violent offenders had impairments in affective processing, were not influenced by social cues (e.g., facial expression), were unable to anticipate punishment, continued make disadvantageous decisions despite awareness of negative consequences, and had reduced neural activity in areas of emotional processing (e.g., fACC) while making decisions. This is in accordance with previous research demonstrating similar impairments in violent offender groups, including low choice consistency, tendencies to focus on immediate versus past outcomes, and poor memory for emotional events (See: Yechiam et al., 2008; Dolan & Fullam, 2005). When examining results of the meta-analysis looking at PD, we found that offenders with PD performed marginally better than controls on tasks of decision-making, but the overall effect was not significant. Due to limited studies being entered into this analysis, such findings should be interpreted with caution. However, it does highlight the need for better quality evidence in order to conduct a larger scale meta-analysis examining these effects in detail.

The neurocognitive deficits identified amongst offenders with mental disorder in the systematic review are broadly similar to those previously demonstrated in non-offenders with equivalent mental disorders. For example, Nishinaka et al. (2016) concluded that offenders with psychotic disorders, including schizophrenia, failed to learn from emotional feedback and that they were less likely to avoid making risky decisions, resulting in poorer performance on the IGT. This is consistent with previous abnormalities detected in clinical samples of patients with schizophrenia (Beninger et al., 2003; Ritter, Meador-Woodruff, & Dalack, 2004; Shurman, Horan, & Nuechterlein, 2005). Similarly, the findings by Jones et al. (2015) and Prehn et al. (2013) that offenders with BPD possess altered emotional decision-making processing and show increased propensity to engage in risk-seeking mistakes respectively, are mirrored by previous studies documenting evidence of risky decision-making in clinical samples (Paret et al., 2017). These findings would suggest that offenders and non-offenders with the same mental disorder cannot easily be distinguished in terms of their decision-making capabilities; however, such a conclusion cannot be drawn given the absence of data directly comparing these two patient groups. It could be that those who offend possess more severe forms of the same or similar deficits in decision-making, or that they are less able to overcome these identified deficits, than their non-offending counterparts. Alternatively, the offending behaviour of criminals with mental disorder may be better explained by extrinsic stressors and factors associated with criminal behaviour; this is supported by how, despite the reported decision-making deficits, offenders with mental disorder did not significantly differ from controls in the overall meta-analysis of IGT performance.

Comparing our review with other reviews, examining decision-making in people with BPD, Paret et al. (2017) identified a similar array of decision-making tasks described in Table 1 (including reversal learning, delay discounting and IGT). Using data from 28 studies, results showed people with BPD achieved lower net gains on the IGT than healthy controls. They also showed an inability to delay reward (using delay discounting task). After exploring moderating factors, it was found current medication, gender, and differences in age between the patient and control group moderated outcome. Moderators were significant with current medication changing group differences, and effect sizes increasing when samples had more female participants. We were unable to explore moderating factors in our PD sub-group analysis due to smaller numbers entered into the analysis. Similarly, only four studies in the review had some representation of female participants. Factors such as gender and current medication are likely to be significant for offender groups and may be influencing the current evidence. Future research should take these factors into consideration, and conduct studies with a better balance of female participants, this is particularly important if conducting research in correctional settings where females are often in the minority. This is also important to help us better understand, assess, and manage the challenges and treatment needs of specific patient groups.

When embarking on this review, we were aware that unpacking the mechanisms underlying decision-making may be complicated by the frequency of comorbidity amongst offender populations (Brooker, Repper, Beverley, Ferriter, & Brewer, 2002). Common characteristics of offender samples in our review included comorbidities such as ASPD and BPD (Prehn et al., 2013), DPD and psychosis (Sedgwick, 2017) and psychosis with mood disorder (Nishinaka et al., 2016). This review has made a start in specifying the problem, unpicking the interactions between, and the effects of, each mental disorder upon offender’s decision-making capabilities, but this continues to represent a significant challenge for future work. Several authors attempted to address the issue by utilizing between-subjects designs. For example, Sedgwick (2017) compared decision-making amongst offenders with DPD (defined as ASPD in the DSM-5) and comorbid psychosis to offenders with DPD only, offenders with psychosis only, and healthy controls. Whilst such approaches are recommended, it is often difficult to prove that group differences are solely due to the presence or absence of particular disorders and no other factors, including individual differences between participants and discrepancies in disorder severity. Larger sample sizes and careful matching criteria are therefore, needed to help minimize the effects of such group differences.

A number of methodological insights were gained by conducting this review. It was notable that all but one of the studies (Jones et al., 2015) included in this review were cross-sectional in nature. Consequently, the temporality of any identified relationships between mental disorder, offending and decision-making cannot be confidently established. This is especially if important if we are to evaluate mental disorder as a potential contributor to the development and perpetuation of decision-making deficits in offenders. The lack of longitudinal data also forbids assessment of how decision-making processes may be altered by passage through the criminal justice system, and how they may change during a person’s rehabilitation and recovery from mental disorder. This is important, since understanding this information could help clinicians to better identify a person’s risk of reoffending at the time of prison release, and the support and interventions required to promote abstinence from further crime. For these reasons, future studies should consider using non-cross-sectional designs. Furthermore, we would encourage the use of more comprehensive control groups. Most studies compared the decision-making functions of offenders with mental disorder to either healthy non-offending controls or criminals with no mental disorder. An exception to this rule was the study by Koenigs et al. (2010) who employed a three-group comparison design exploring gradients of psychopathy (primary. Vs secondary. Vs non-psychopaths). This study did find significant differences in scores on the UG between primary and secondary psychopaths. Future studies should select control groups carefully, incorporating non-offenders with mental disorder. This would allow us to better unpick the effects of mental disorder and other factors associated with offending upon criminal decision-making and perhaps help us to understand why some individuals with a given mental disorder commit crimes, whilst others do not. Following on from this, such comparisons could also help clinicians to identify what (if any) decision-making deficits are likely to improve following successful treatment of a person’s underlying mental disorder, and which ones may exist independently to this across offender groups.

This review highlighted a further limitation of research in this area, namely the heterogeneity of studies assessing decision-making in offender populations, despite many studies using comparable decision-making outcome measures, such as the IGT. The issue was previously noted by Hughes et al. (2016) in their review of decision-making in psychopathy, with our review providing further support. The major differences identified in this review include differences in study setting (e.g., prison vs. community vs. probation) and control group definition (e.g., offenders with no mental disorder vs. non-offending healthy controls). Such differences make it difficult to draw meaningful conclusions since it is currently unclear what impact these inconsistencies would have on each studies data.

### Future Directions

Based on analysis of individual studies, it would be useful to understand which sub-groups of offenders have impaired decision-making so that this can be identified, assessed and treated in the hope of preventing further criminal behaviour. Research with mixed samples of offenders with different types of co-morbidity (and potential confounding issues such as brain injury) may be influencing results and making meta-analysis difficult to conduct and interpret. It may also be useful to understand other independent variables such as disorder type so that any psychological interventions aimed at improving decision-making are tailored to the specific needs and challenges of each patient group. Furthermore, studies included in this review excluded offenders with TBI. Therefore, we excluded studies involving decision making in offenders with TBI. Such studies merit a standalone review.

## Conclusion

Individual studies suggest offenders with mental disorder make poorer decision compared to controls, and the means of offenders were qualitatively lower on the IGT and SAT when entered into a meta-analysis, but the pooled effect was not statistically significant. The quality of the current evidence is mixed, there remains no single definition of decision-making, and characteristics of offenders and study design makes it difficult to draw clear conclusions. This review has attempted to decide on a single definition of decision-making, bring together tasks that can be considered to assess this, and provide meaningful future directions for researchers working in this area.

## Electronic supplementary material

ESM 1 (DOCX 58 kb)

ESM 2 (DOCX 23 kb)
