# Supplementary material for: A Systematic Review and Meta-Analysis of Decision-Making in Offender Populations with Mental Disorder
Source: Neuropsychol Rev. 2019 Feb 23;29(2):244–58. doi: 10.1007/s11065-018-09397-x (PMC6560009; doi:10.1007/s11065-018-09397-x)

**Online Figure 1. Funnel plot for all studies included in meta-analysis.**


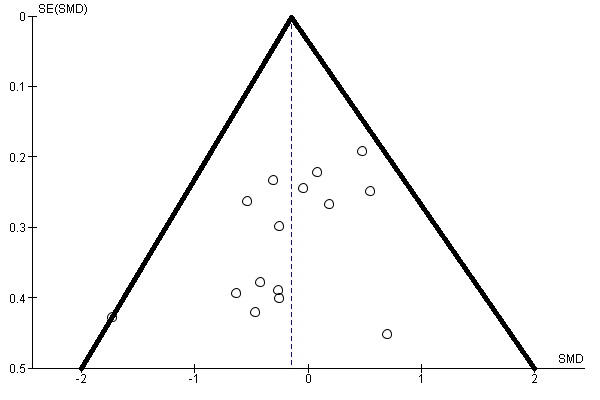


Since some of the studies in the meta-analysis are small and/or showing no statistically significant effects then publication bias can lead to the observed asymmetrical appearance of the funnel plot. It is important to realize that publication bias is only one of a number of possible explanations for funnel-plot asymmetry; trials of lower quality yield exaggerated estimates of treatment effects, smaller studies are, on average, conducted and analysed with less methodological rigor than larger studies, so asymmetry may also result from the overestimation of treatment effects in smaller studies of lower methodological quality. Intensity of intervention along with heterogeneity due to poor choice of effect measure can be a possibility as well. The more pronounced the asymmetry, the more likely it is that the amount of bias will be substantial. Since this funnel shows some symmetry it seems appropriate that this is not completely asymmetrical suggesting minimal-moderate bias.

The Begg and Mazumdar (1994) adjusted rank correlation test is a direct statistical referent to the funnel chart. Both the test and the funnel have less power to detect publication bias. The test for publication bias is determined if there is a significant correlation between the effect estimates and their variances. When this test was used on the data, a P value of 0.225 was obtained. The Egger et al, (1997) regression asymmetry test and the regression asymmetry plot suggest publication bias more regularly than Begg’s approach. The Egger test detects funnel plot asymmetry by determining if the intercept deviates significantly from zero in a regression of the standardised effect estimates against their precision. Egger's test for bias gave a P value of 0.09.

**Online Figure 2. Meta-analysis decision-making in Psychopathic and PD offenders using IGT**


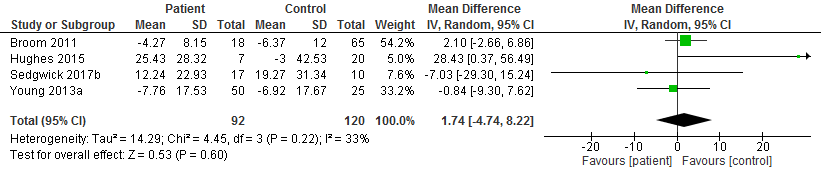


**Online Figure 3. Meta-analysis decision-making in DWI offenders using IGT**


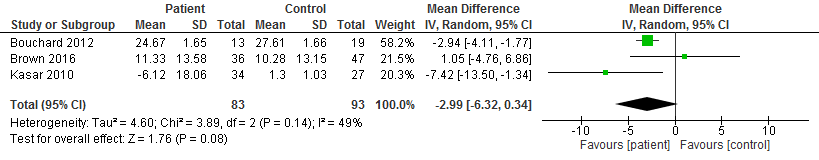

Supplement: Supplementary file 1 — (DOCX 58 kb) [file 11065_2018_9397_MOESM1_ESM.docx]
