# Supplementary material for: A Systematic Review and Meta-Analysis of Decision-Making in Offender Populations with Mental Disorder
Source: Neuropsychol Rev. 2019 Feb 23;29(2):244–58. doi: 10.1007/s11065-018-09397-x (PMC6560009; doi:10.1007/s11065-018-09397-x)
Supplement: Supplementary file 2 — (DOCX 23 kb) [file 11065_2018_9397_MOESM2_ESM.docx]

**Online Table 1. List of excluded studies and reasons for exclusion**

| Study | Study Description and Reason for Exclusion |
| --- | --- |
| AHMED  (2015) | This study examined the effects of neurocognitive functions and cognitive training on aggression and violent offending in persons with schizophrenia and schizoaffective disorder. It was excluded since the full text article could not be accessed. |
| BASS  (2010)# | This study examined decision-making and aggression in forensic psychiatric inpatients. It was excluded due to the lack of a control group. |
| BELL  (2009) | This study examined psychopathy, PD, criminality, and neurocognitive performance in a large cohort of male and female prisoners across England and Wales. It was excluded due to the lack of a control group. |
| BESTERCZEY  (2007) | This study investigated disadvantageous decision-making and its relationship to adverse outcomes a sample of ex-offenders, including some with substance abuse, who were at risk of repeat offending or relapsing. It was excluded since the full text article could not be accessed. |
| BLACKWOOD  (2012) | This study assessed structural and functional brain differences between violent offenders with antisocial personality disorder (ASPD) and psychopathy, violent offenders with ASPD but not psychopathy, and healthy non-offending controls. It was excluded because the full text article could not be accessed. |
| BROWN  (2017) | This study examined the course of cognition in mentally ill offenders and the implications for risk of violence. It was excluded due to the lack of a control group. |
| CHEN  (2015) | This study assessed predictors of relapse and drop-out during a 12-week relapse prevention program for Methamphetamine users sentenced by the criminal justice system. It was excluded due to the lack of a control group. |
| DOLAN  (2008) | This study examined the relationships between family history of substance abuse, executive function, impaired decision-making, and current substance abuse in a sample of substance-dependent inpatients. It was excluded because it was unclear what proportion of the sample, if any, had been convicted of crimes relating to their substance-abuse and hence, the offending status of the sample was unknown. |
| FISHBEIN  (1998) # | This study compared neuropsychological functioning and its relationship to violence between pre-release inmates with and without substance abuse. It was excluded since the data was unpublished and unavailable. |
| HANSEN  (2015) | This study examined the effects of a long-term fatty fish intervention on executive functioning in male forensic inpatients. It was excluded due to the lack of a control group. |
| KANZ  (2005) | This study explored relationships between self-control, decision-making and criminal risk in offenders at the Iowa Medical and Classification Centre. It was excluded since the full text article could not be accessed. |
| KONOMI  (2008) | This study examined the neuropsychological and personality functions of forensic and non-forensic psychiatric inpatients and assessed their relations to violent behaviour. It was excluded since the full text article could not be accessed. |
| LEVI  (2005) | This study examined the personalities and neuropsychological functions, including decision-making capabilities, of primarily predatory, irritable and non-aggressive offenders. It was excluded since the full text article could not be accessed. |
| O’HANLON (2012) | This study examined frontal lobe deficits and anger as violence risk markers for males with major mental illness in a high-secure hospital. It was excluded due to the lack of a control group. |
| PRENDERGAST  (2002) | This study examined psychosocial changes (including decision-making) in offenders during treatment and drew comparisons between those admitted voluntarily and involuntarily. It was excluded due to the use of self-report measures instead of an objective decision-making task. |
| ROBINSON  (1981) | This study explored the determinants of offender’s decisions to drive or not to drive during their disqualification from driving period. It was excluded due to the use of a self-report questionnaire and hence, the lack of an objective decision-making task. |
| SCHMOLL  (2012) | This study draws together findings on decision-making in psychopathy, with a particular focus on legal responsibility. It was excluded since the full text article was not available in English. |
| SEON  (2008) | This study compared the decision-making abilities of forensic patients who had been found not criminally responsible for their crime to community mental health inpatients. It was excluded since the full text article could not be accessed. |
| SHIRAI  (2014) | This study explored relationships between neuropsychological measures, including risky decision-making, criminal and substance use history in a sample of ex-offenders. It was excluded since the full text article could not be accessed. |
| SNOWDEN (2017) | This study examined the relationship between risk-taking and psychopathy amongst male offenders and undergraduate students. It was excluded because it was unclear what proportion, if any, of the male offenders suffered from mental illness; higher levels of psychopathy, as measured by self-report, were in fact demonstrated in the control group. |
| SUCHY  (2008) | This study compared neurocognitive functions between paedophilic and non-paedophilic child molesters, and community controls. It was able to examine decision time by using simple and complex choice reaction time tasks, but did not include any measures of decision-making quality. This study was therefore excluded due to the lack of an appropriate decision-making task. |
| THORELL  (2017) | This study compared delay-related behaviours between adults with ADHD and clinical and non-clinical controls. It also related these to measures of functional impairments, including criminality. It was excluded since the self-report measure of delay discounting did not significantly correlate with the laboratory measure, and only the former was used in relation to the assessment of criminality. |
| YECHIAM  (2008) | This study examined the cognitive processes of criminal offenders incarcerated for various crimes. It was excluded because it was unclear what proportion, if any, of the offenders suffered from mental illness. |

# = Identified through hand searching.

**Online Table 2. Quality assessment of included studies using National Heart, Lung and Blood Institute Quality Assessment Tool for Observational Cohort and Cross-Sectional Studies**

| Study | Research question/objectives | Study population and size | Timeframe/follow up | Exposure Measures | Outcome Measures | Confounding | Overall quality (max 14) |
| --- | --- | --- | --- | --- | --- | --- | --- |
| BALIOUSIS (2014) | +++ | +++ | + | ++ | +++ | +++ | +++  Good |
| BESZTERCZEY (2013) | +++ | +++ | + | ++ | ++ | + | ++  Moderate |
| BOUCHARD (2011)* | ++ | +++ | + | ++ | ++ | ++ | ++  Moderate |
| BROOM  (2011)* | ++ | + | + | ++ | ++ | ++ | ++  Moderate |
| BROWN  (2016)* | ++ | +++ | + | ++ | ++ | +++ | +++  Good |
| DEBRITO  (2013) | ++ | ++ | + | ++ | ++ | ++ | ++  Moderate |
| GULEC  (2007)* | ++ | ++ | + | ++ | ++ | + | +  Poor |
| HUGHES  (2015)* | ++ | +++ | + | ++ | ++ | ++ | ++  Moderate |
| JONES  (2015) | ++ | + | ++ | +++ | ++ | ++ | ++  Moderate |
| KIRKPATRICK (2007) | ++ | ++ | + | ++ | ++ | ++ | ++  Moderate |
| KOLLA  (2015) | ++ | ++ | + | ++ | ++ | +++ | ++  Moderate |
| KOENIGS  (2010) | ++ | ++ | + | ++ | ++ | ++ | ++  Moderate |
| KUOKKANEN (2016) | ++ | +++ | + | ++ | ++ | ++ | ++  Moderate |
| LY  (2016) | ++ | +++ | + | ++ | ++ | ++ | ++  Moderate |
| KASAR  (2010)* | ++ | ++ | + | ++ | ++ | +++ | ++  Moderate |
| NISHINAKA (2016)* | ++ | +++ | + | ++ | ++ | ++ | ++  Moderate |
| PREHN  (2012) | ++ | ++ | + | ++ | ++ | ++ | ++  Moderate |
| RADKE  (2013) | ++ | ++ | + | ++ | ++ | ++ | ++  Moderate |
| RODRIGUEZ (2017)* | +++ | ++ | + | ++ | ++ | ++ | ++  Moderate |
| SEDGWICK (2017)* | +++ | ++ | + | +++ | ++ | ++ | ++  Moderate |
| WELLS  (2009) | ++ | ++ | + | ++ | + | + | +  Poor |
| YOUNG  (2012)* | ++ | +++ | + | ++ | ++ | ++ | ++  Moderate |
| YOUNG  (2013)* | ++ | ++ | + | ++ | +++ | ++ | ++  Moderate |

+ = Poor, ++ = Moderate, +++ = Good

*Studies entered into meta-analysis.

<https://www.nhlbi.nih.gov/health-topics/study-quality-assessment-tools>
